# Nutritional correlates and dynamics of diabetes in the Nile rat (*Arvicanthis niloticus*): a novel model for diet-induced type 2 diabetes and the metabolic syndrome

**DOI:** 10.1186/1743-7075-7-29

**Published:** 2010-04-15

**Authors:** Fadi Chaabo, Andrzej Pronczuk, Ekaterina Maslova, KC Hayes

**Affiliations:** 1 Foster Biomedical Research Laboratory, Brandeis University, Waltham, MA, USA, 02454

## Abstract

**Background:**

The prevalence of Metabolic Syndrome and related chronic diseases, among them non-insulin-dependent (type 2) diabetes mellitus, are on the rise in the United States and throughout the world. Animal models that respond to environmental stressors, such as diet, are useful for investigating the outcome and development of these related diseases.

**Objective:**

Within this context, growth and energy relationships were characterized in the Nile rat, an exotic African rodent, as a potential animal model for diet-induced type 2 diabetes mellitus and Metabolic Syndrome.

**Methods:**

Compiled data from several studies established the relationship between age, body weight gain (including abdominal adiposity), food and water consumption, and blood glucose levels as determinants of diabetes in male and female Nile rats. Glucose Tolerance Testing, insulin, HbA1c, blood pressure measurements and plasma lipids further characterized the diabetes in relation to criteria of the Metabolic Syndrome, while diet modification with high-fat, low-fiber or food restriction attempted to modulate the disease.

**Results:**

The Nile rat fed lab chow demonstrates signs of the Metabolic Syndrome that evolve into diet-induced non-insulin-dependent (type 2) diabetes mellitus characterized by hyperinsulinemia with rising blood glucose (insulin resistance), abdominal adiposity, and impaired glucose clearance that precedes increased food and water intake, as well as elevated HbA1c, marked elevation in plasma triglycerides and cholesterol, microalbuminuria, and hypertension. Males are more prone than females with rapid progression to diabetes depending on the challenge diet. In males diabetes segregated into early-onset and late-onset groups, the former related to more rapid growth and greater growth efficiency for the calories consumed. Interestingly, no correlation was found between blood glucose and body mass index (overall adiposity) in older male Nile rats in long term studies, whereas blood glucose and the perirenal fat pad, as well as liver and kidney weight, were positively related to early-onset diabetes. Rats weaned early (4-5 wks) and challenged with a high-fat Western-type diet developed diabetes faster, and body fat accumulation was more apparent, whereas food restriction curtailed it.

**Conclusion:**

The Nile rat fed typical rodent diets develops hyperinsulinemia that precedes hyperglycemia (insulin resistance) leading to diet-induced type 2 diabetes associated with hypertriglyceridemia, hypercholesterolemia, and hypertension. Dietary modulation affected growth rate (weight gain and central adiposity) to impact disease progression. This rodent model represents a novel system of gene-diet interactions affecting energy utilization that can provide insight into the prevention and treatment of the type 2 diabetes and Metabolic Syndrome.

## Background

The Nile rat, aka the African grass rat (*Arvicanthis niloticus*), is gerbil-like and native to the Nile River delta and across most of sub-Saharan Africa where it lives in underground burrows and feeds mainly on vegetative plants and grass seeds [1]. It was imported to the US in 1990s to establish breeding colonies as a diurnal model for studies of sleep and circadian rhythm [2]. However, such studies are often complicated by diabetes that develops slowly in many animals within the first months of life.

The Sand rat (*Psammomys obesus*) is a similar gerbil-like rodent native to North Africa and the Near East that has been investigated for energy-dependent diabetes and serves as a valuable comparison [3-6]. It has a similar background of food scarcity in its natural habitat; but when fed a laboratory chow diet in captivity, it experiences rapid onset of diabetes, a disease that has not been observed among sand rats or Nile rats in the wild. Two lines of sand rats have been bred by selection, one diabetes-prone and one somewhat diabetes-resistant, with the former demonstrating greater conversion of diet energy efficiency into body weight gain than the latter [3].

With growing concern over the worldwide epidemic in diabetes [7], the African grass rat was examined as a potential model of type 2 diabetes that might allow more detailed nutritional studies of a diet-induced diabetes similar to that in humans. Since type 2 diabetes in humans is associated with several parameters that are referred to as the Metabolic Syndrome and the Insulin Resistance Syndrome [8,9], these parameters became the focus of our studies. This report provides the first details concerning various aspects of husbandry, energy requirements, as well as the potential for nutritional and age-related modulation of the disease.

## Methods

### Breeding and weaning

In May 2005 six breeding pairs of Nile rats were obtained from Michigan State University (courtesy of Dr. Laura Smale, Psychology Dept, who initially encountered the diabetes). These breeding pairs were maintained at Brandeis University in air-conditioned rooms with a 12 h light cycle (temperature 68-72F, humidity 40-60%). All breeders were fed ad libitum a typical laboratory chow (Lab Diet #5008, 3.3 kcal/g, PMI Nutrition International, Brentwood, MO) and water. Sixty-four breeding pairs were added over the next 4 years, with selected rats evaluated for diabetes from different experiments and from miscellaneous groups not on nutritional study. Approximately 1100 pups were weaned at 3 and 12 weeks of age over this period and fed a second laboratory chow (Lab Diet #5020, 3.6 kcal/g) ad libitum with water. At different ages rats were assessed for random (nonfasting during the day) or fasting blood glucose after overnight 16-hour food restriction. They were then placed in individual cages and evaluated further. All experiments were approved by the Brandeis IACUC for experimentation with animals.

### Handling

Nile rats are alert, nervous and somewhat skittish rodents that quickly dash into their 2" PVC tubing when alarmed. Social animals living in burrows in their natural habitat, the Nile rats thrives in the comfort of parents and/or siblings. Isolation in individual cages may contribute to its slightly aggressive nature, although this dissipates with time and handling. Chewing of tails and ears occurs occasionally in grouped rats, which can make tail bleeds difficult if scaring occurs. Barbering has been observed in individuals, but especially when housed in groups, and can be either focal or diffuse in nature. Gregarious, defensive and biting when handled, capture of the Nile rat is readily accomplished by gloved hand. Gently transferring rats from cage to cage is achieved with rats hiding in their PVC tubes, or by grasping tails, either with gloved hand or using long forceps with tips covered by rubber tubing. When threatened or frightened, young rats may try to escape when handled.

### Body weight

Weight was measured either with a rat in its PVC tube (that had been previously tared) or when under light anesthesia using a 50-50 oxygen and carbon dioxide gas mixture funneled into the tube.

### Body Mass Index (BMI) and Lean Mass Index (LMI)

The BMI and LMI for Nile rats were measured to determine whether body mass affected diabetic status. BMI was calculated by dividing the body weight in kilograms by the length (head to base of tail) in meters squared. LMI was calculated the same way, except the carcass weight (after all organs, brain, and fat depots were removed) was used instead of the body weight.

### Blood glucose

Fasted rats were deprived of food and given only water for 16 h overnight, while random blood glucose was measured in non-fasted animals during the day (usually between 11 am and 3 pm). To minimize handling and excitement, rats were anesthetized when quietly hiding in their PVC tubes by directing a 50-50 mixture of O_2_+CO_2 _into the tube for 15-30 seconds. Blood glucose was then measured from tail blood using an Elite XL glucometer (Bayer Co., Elhart, IN). The rat was returned to its cage to awaken within 1 min.

### Plasma triglycerides and total cholesterol

Blood samples were collected from the tail (30-70 μl) or by cardiac puncture (100-200 μl with 30# needle) under O_2_/CO_2 _anesthesia and placed in plastic vials moistened with EDTA. Plasma triglycerides (TG) and total cholesterol (TC) were determined spectrophotometrically using Infinity™ kits (Thermo Fisher Scientific Inc., Middletown, VA, TG ref # TR22421, TC ref # TR13421).

### Liver lipids

Liver lipids (TG and TC) were extracted from 0.1 g of tissue ground with 4 g of sodium sulfate using a 2:1 chloroform:methanol solution. Total extract was combined and dried under nitrogen and redissolved in 1 ml of chloroform. An aliquot (10-20 μl) of each sample was dried under nitrogen and dissolved in 50 μl of Triton X-100 and chloroform (1:1 by volume). The solution was dried extensively to remove chloroform, and TG and TC determined using the appropriate Infinity™ kit.

### IpGlucose Tolerance Test (ipGTT)

Rats were fasted 16 h (overnight) and injected intraperitoneally with a glucose solution in saline at 2.5 g/kg body weight. Blood glucose was measured from tail bleeds before glucose injection (0 time) and after 1, 3 and 5 h using an Elite XL glucometer.

### Hemoglobin A1c (HbA1c)

The animals were deeply anesthetized with 50%/50% O_2_/CO_2_, and blood was collected by cardiac puncture using EDTA-wetted insulin syringes. Glycated hemoglobin (GHb) was measured by GLYCO-Tek affinity column kit (Helena Laboratories, Beaumont, Texas). HbA1c was calculated using the equation provided by the company.

### Insulin Enzyme-Linked Immunosorbent Assay for Insulin (ELISA)

Plasma insulin was determined with an enzyme-linked immunosorbent assay (ELISA) kit for rat/mouse insulin (Linco Research, Millepore, Billerica, MA, catnr. EZRMI-13K), according to the manufacturers' protocols.

### Males vs females (Expt 1)

To examine sex differences in diabetic response, a group of 13 males and 10 females was fed lab chow #5020 ad libitum with water in groups of four animals after weaning at 5 wks until housed separately from 11 wks to 44 wks. Daily food and water intake were recorded, and body weight gain and random blood glucose were measured at different time points.

### Blood glucose determinants (Expt 2)

For an overview on the relationship between growth and diabetes progression, glucose was assessed in a larger pool of approximately 100 male and 60 female individually housed Nile rats fed lab chow #5020 and water *ad libitum *weaned at the age between 6 and 12 wks by measuring both random or fasting tail bloods in cross-sections of rats up to 36 wk of age.

### Early-onset, late-onset study (Expt 3)

To describe individual Nile rat growth dynamics and diabetes onset in detail, 11 male eight-wk old rats were fed a high-fat, low-fiber Western-type purified diet (CHO:Fat:Protein, energy ratio of 40:43:17, 4.5 kcal/g, Table 1, Hayes-Cathcart vitamin mix [10]) for 24 wks. After the initial 12 wks on diet, rats were categorized as either early-onset (n = 6), or late-onset diabetic (n = 5) based on a random blood glucose >150 mg/dl or <150 mg/dl, respectively. Parameters continued to be monitored until 32 wk of age.

**Table 1:** Composition of purified diets^1^

INGREDIENTS	Diet	
	
	Mediterranean-type	Western-type
	(Low-fat/high-fiber)	(High-fat/low-fiber)
**CHO:Fat:Prot %en**	**69:15:16**	**40:43:17**
**kcal/g**	**3.5**	**4.5**

	g/Kg	
Casein	20	80
Lactalbumin	20	80
Oatmeal (7.5%fat)	733	200
Wheatbran (4.25% fat)	70	0
Dextrose	0	170
Cornstarch	60	170
Cellulose	44	31
(Total fiber)^2^	(147)	(51)
		
Fat (Sats:Monos:Polys)	(3:5:7)	(19:18:6)
		
Milk fat (chol stripped)		42
Tallow (chol stripped)		97
Lard		31
Soybean oil		30
(Total fat)^2^	(58)	(15)
		
Mineral mix (Ausman - Hayes)^3^	40	52
		
Vitamin mix (Hayes - Cathcart)^4^	11	14
		
Choline chloride	2	3

^1 ^Diets which were prepared in the lab were fed to Nile rats as cake, prepared by with holding from the formulation 60 g/kg cornstarch premixed into 800 mL of simmering water before addition to the remaining ingredients

^2^Includes fiber or fat in oatmeal and wheatbran

^3^Ausman-Hayes mineral mix # 210077, Diets, Inc., Bethlehem, PA

^4 ^Hayes-Cathcart vitamin mix

### Body fat and diabetes (Expt 4)

In order to elucidate the relation of body fat pools to stages of diabetes, 20 male rats were fed lab chow #5020 and followed individually from separation at 12 wks to 7 mo (10 rats) or 11 mo of age (10 rats). Random blood glucose was measured at 7 or 11 mo. Fasting terminal blood glucose and plasma lipids, as well as organ weights (including fat pads), were assessed.

### Insulin resistance and diabetes categories (Expt 5)

To map diabetes onset and progression from an early age, 83 male Nile rats were weaned (separated) and challenged with the Western-type diet (Table 1). Rats were weaned (separated from parents) at either 4-5 wks (fed Western-type diet for 2 wks), or at 6-7 wks (fed Western-type diet for 4 wks), or at 8-9 wks (fed Western-type diet for 12 wks) in order to establish different windows for evaluation of diabetes. Insulin concentration, random blood glucose, body weight and plasma lipids were analyzed when sacrificed after 2, 4, or 12 wks of challenge. The rats were then categorized according the progression of the disease in four stages classified as A, B, C and D [11]. Stage A was characterized by low plasma insulin (<3.5 ng/ml) and low random blood glucose (<150 mg/dl); stage B (hyperinsulinemia) by elevated insulin (>3.5 ng/ml) and low random blood glucose (<150 mg/dl); stage C (diabetes) by both elevated insulin (>3.5 ng/ml) and random blood glucose (>150 mg/dl); while stage D (beta-cell failure) represented declining insulin (<3.5 ng/ml) and elevated random blood glucose (>150 mg/dl). Collectively they reflected stages of diabetes from insulin resistance to hyperglycemia, followed eventually by beta-cell failure and insulin depletion. Stage E is characterized by return to low insulin (<3.5 ng/ml) and low random blood glucose (<150 mg/dl) observed in the end stage of the disease (ie. after having passed through all earlier stages and they cease to eat). None of the animals analyzed in these experiments reached this stage, which is encountered only rarely.

### Early weaning effect on diabetic categories (Expt 6)

To further assess diabetes categories and organ changes following early weaning, 8 male Nile rats were monitored closely after weaning at 5 wks, while fed our high-fat, low-fiber Western-type diet (Table 1). After 4 wks of diet challenge, the 8 rats were killed, and subsequently categorized into two groups of 4 (early-onset and late-onset diabetes) based on their random blood glucose, plasma lipids, insulin, and HbA1c. Food and water intake, as well as body weight were monitored.

### Effect of high-fat versus low-fat diets (Expt 7)

To examine possible dietary impact on diabetes, rats were challenged with the Western-type diet or a low-fat, high-fiber Mediterranean-type diet (Table 1) to assess the impact on progression of diabetes. Twelve 5-wk old males were divided into three groups of 4 and fed for 24 wks either of these two purified diets or a lab chow (ProLab, RMH #3000, energy ratio 60:14:26, 3.2 kcal/g). Food and water consumption was measured twice weekly for 24 wks. Body weight and tail random blood glucose were measured at the beginning of the experiment and after 24 wks. The rats were anesthetized, and final fasting blood glucose was measured before exsanguination. The internal organs and different fat pads were collected and weighed, and plasma as well as liver were analyzed for TG and TC. Additionally, at about one week before termination an intraperitoneal glucose tolerance test (ipGTT) was conducted to evaluate the effect of caloric density on the development of diabetes.

### Calorie restriction study (Expt 8)

A food restriction study was conducted to determine its effect on diabetes. Eleven 20-wk old Nile rats (6 males and 5 females) were assigned to 2 groups with ample access to water and fed lab chow #5020 or chow restricted to 75% of *ad libitum *for another 18 wks. Body weight and blood glucose were measured at the beginning, mid-point and end point of the study. An ipGTT was assessed by tail bleeds, followed by terminal exsanguination under anesthesia to collect plasma for additional measures. Internal organs and selected fat pads were excised and weighed.

### Blood pressure (Expt 9)

To evaluate blood pressure, 13 male 8-wk old Nile rats were weaned to lab chow (Lab Diet, #5020). Blood pressure was measured 4 times between 27 wk to 43 wk of age using an all-cuff noninvasive IITC Life Science manual inflation amplifier Model 29 (NIBP Amplifier, Woodland Hills, CA. 91367).

To calibrate the cuff the temperature chamber was set to 32°C and the cuff and cables placed inside. The rat was briefly anesthetized with 50%/50% O_2_/CO_2 _to place it without struggle in the restrainer inside the temperature chamber. A plastic collar (1 cm × 0.5 cm) was fitted inside the cuff to better adapt the tail diameter to sensor diameter. Preliminary tests revealed the effectiveness of this modification with improved signal recordings. Once the measurements stabilized, the cuff was inflated to 100 mmHg, then 150 mmHg or more to assure correct readings. Measurements were repeated 3-5 times and the average of this values was taken. Growth dynamics and fasting blood glucose were also examined. After the initial 14 wks on lab chow (Lab Diet, #5020), the 13 rats were categorized with either early-onset (n = 3), or late-onset diabetes (n = 10) based on a fasting blood glucose >110 mg/dl or <110 mg/dl, respectively. At the age of 43 wks the animals were exsanguinated from the heart to assess their lipid and insulin profiles.

### Growth efficiency

By measuring daily food intake and body weight gain over a set period of growth, it was possible to calculate growth efficiency as the number of calories needed per gram of body weight gain. Thus, the highest growth efficiency represented the fewest calories consumed per weight gained.

### Statistical analysis

Statistical analysis was performed using the Super ANOVA statistical software (Abacus Concepts. Inc, Berkeley, CA). Corrected Students T-test for unequal variances (p < 0.05), or one-way ANOVA with a posthoc Fischer's PLSD test (p < 0.05), were conducted where appropriate to study design. Z-test was used for correlations.

## Results

### General colony dynamics, Expts 1 and 2

#### Nile rat growth (Expt 1)

**Figure 1:**
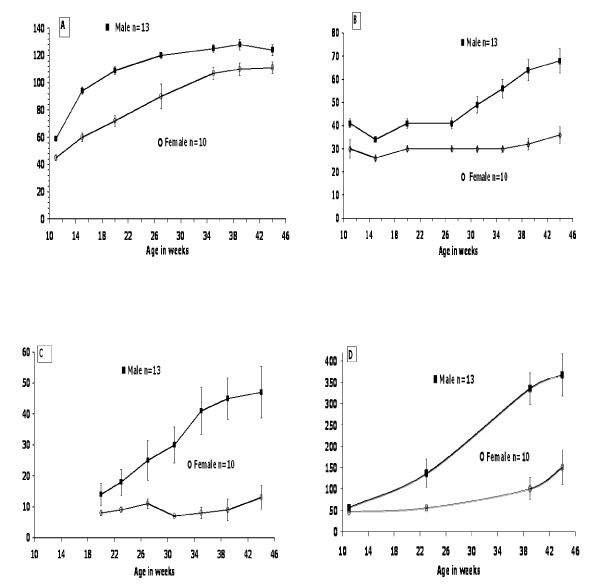
**(Expt 1)**. Nile rat growth dynamics for 13 males and 10 females fed lab chow (Lab Diet, #5020). Body weight change (A), energy (B) and water intake (C) and random blood glucose (D) in male and female Nile rats were measured periodically (mean ± SEM) between 11 and 44 wk of age.

Figure 1 depicts the growth rate, energy intake, water intake, and random blood glucose in 23 rats (13 males and 10 females) that had been housed in groups of 4 until the start of the study at 11 wks, when they were housed individually. In general, the average weight of 8-wk old weanlings is about 50 g for females and 60 g for males. These 23 rats were monitored at monthly intervals for 8 mo, ie. between 11 and 44 wks of age. Males grew faster, gaining 80% of their total weight between 11-20 wks, while females gained only 40% of their final weight in that period. Males reached a final body weight of approximately 130 g at 40 wks, while females finally weighed about 110 g. In general, Nile rats that survive beyond 1 year eventually die due to consequences of hyperglycemia, hyperlipidemia with high triglycerides, elevated cholesterol and hypertension. Elevated VLDL and LDL with depressed HDL (unpublished data) and renal dysfunction with microalbuminuria along with cataracts and other complications associated with progressive diabetes were also observed (data not shown).

#### Energy and water intake

Figure 1B displays the energy intake of the same 23 Nile rats. While females only increased caloric intake slightly over time, males increased food intake substantially after 27 wks *following *diabetes onset. Food intake was greater for males than females at every time point. At 39 wks males (mostly diabetic by now) averaged 64 kcal/d compared to 32 kcal/d for females (mostly nondiabetic). Figure 1C indicates that water consumption increased in males as food intake (and blood glucose) increased. Normal females drank about 10 ml/d, while normal males drank about 15 ml/d. Male Nile rats with fasting blood glucose over 350 mg/dl drank as much as 70 ml/day.

#### Random blood glucose

Random blood glucose was measured at 23, 39 and 44 wk of age in these same rats (Figure. 1D). Males had higher blood glucose at all time points, almost tripling between 23 wks and 39 wks from 137 mg/dl to 336 mg/dl. In females glucose only doubled from 56 mg/dl to 101 mg/dl during the same 16 wk interval. At 44 wk random glucose in male rats had increased slightly to 368 mg/dl, while females had increased substantially to 151 mg/dl.

### Blood glucose determinants (Expt 2)

**Figure 2:**
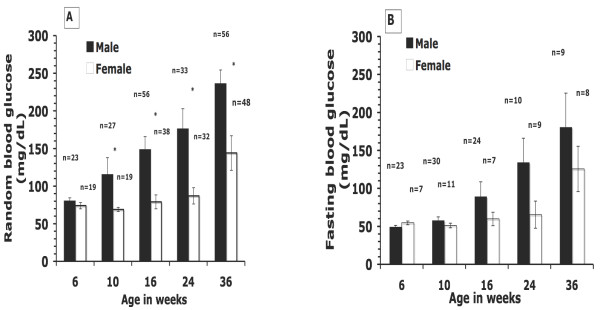
**(Expt 2)**. Blood glucose distribution is depicted for a cross-section of male and female Nile rats fed chow (#5020). Random blood glucose (A) or fasting blood glucose (B) was measured at different ages (6-36 wks). Observations, n, varied at each time period. Values are mean ± SEM. *Significantly different (P < 0.05).

Fasting and random blood glucose were measured in various experiments to determine the progression of diabetes in typical rats fed chow (Figure. 2A,B). Both values increased with age in both sexes, with males exceeding females at all time points. Both random and fasting blood glucose typically averaged between 40-60 mg/dl for 4-6-wk old Nile rats of either gender, with the lower values associated with the youngest rats. Random blood glucose initially increased as early as 10 wks in some males, with fasting values following suit; and males diverged considerably from females as they aged. The increase in males was typically steady and dramatic by 24-36 wks, with random values averaging about 240 mg/dl for males and 140 mg/dl for females in that time period. In rats surviving beyond one year, weight loss was commonly observed as diabetes progressed, often associated with decreased food and water intake at the end stage of the disease (data not shown).

### Individual blood glucose (diabetes) dynamics (Expt 3)

**Figure 3:**
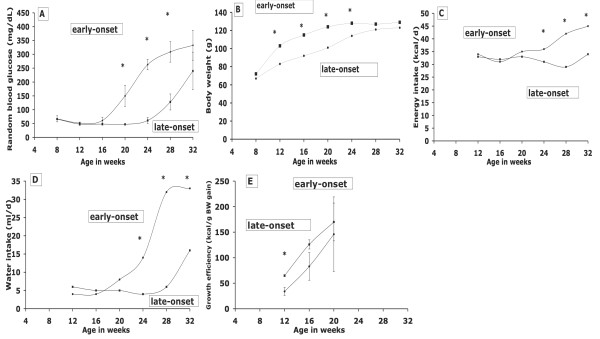
**(Expt 3)**. Dynamics of random blood glucose, body weight, food and water intake and energy efficiency for two groups of male Nile rats with early or late onset to diabetes. Eleven 8 wk-old male Nile rats were fed Western-type diet for 24 wks and categorized as either early-onset (n = 6) or late-onset (n = 5) based on random blood glucose. Random blood glucose (A), body weight (B), caloric intake (C), water intake (D) and growth efficiency (E) were measured monthly for both onset groups over a period of 6 mo. Note that weight gain preceded glucose elevation, especially in the early-onset group. Values are mean± SEM. *Significantly different (p < 0.05).

The wide variation in growth observed among male Nile rats in preliminary studies raised the question whether growth rate *per se *influenced diabetes onset. Thus, in experiment 3 the *random *blood glucose in a typical cohort of 11 male Nile rats was measured monthly between 8 and 32 wk of age, comparing glucose to growth rate as well as growth efficiency based on energy intake (Figure. 3). Two distinct growth patterns were associated with blood glucose. At 24 wk of age an early-onset diabetic group (random glucose >150 mg/dl, n = 6) was observed to have more rapid growth. This subset was retrospectively found to have elevated glucose at 20 wk of age, which was already significantly higher than the late-onset group (n = 5). Blood glucose remained normal in the latter group until 24 wks, whereupon it began to rise to finally approximate that of the early-onset group by the end of study at 32 wks (Figure. 3A). In other words, diabetes was delayed about 10-12 wks in the late-onset group.

Body weight data revealed that the early-onset group gained 75% (to 115 g) of final weight by 16 wks (Figure. 3B), when random blood glucose began to increase (Figure. 3A). By contrast, the late-onset group gained significantly less at only 44% (to 92 g) by 16 wks. At 24 wks the early-onset group had reached 98% final weight (128 g) with an elevated random blood glucose (263 mg/dl), whereas the late-onset group was at 84% final weight (114 g) and 61 mg/dl, respectively. In fact, during weeks 8-12 the early-onset group gained twice the weight of late-onset rats, while consuming equal calories (Table S1, Additional file 1). Thus, growth efficiency (per calorie consumed) in this period was double that of early-onset rats (p < 0.05), even though blood glucose was still low and identical between groups. However, by 16-20 wks when weight gain slowed in early-onset rats, their blood glucose had tripled. On the other hand, the blood glucose rose about 10-12 wks later in the late-onset group, *after *their weight gain accelerated and body weight had increased and plateaued. The enhanced growth efficiency of early-onset rats disappeared by the end of study, when both groups were equally diabetic and weight gain (growth) had stabilized at nearly equal body weights. Since growth was essentially over, food energy reflected weight maintenance and ceased to be relevant for estimating growth efficiency (Figure. 3E). Differences were more pronounced for water intake (Figure. 3C,D).

**Figure 4:**
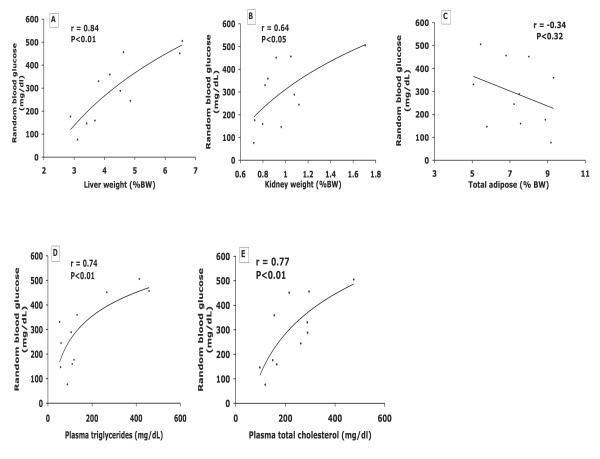
**(Expt 3)**. Correlations between random blood glucose and liver, kidney, adipose weight (expressed as %BW) and plasma lipids for 11 male Nile rats. Correlation between blood glucose and liver (A), and kidney (B), and total adipose (C), and TG (D), and TC (E). The slightly inverse trend for adipose likely reflects the probability that fat disappears as diabetes advances.

To assess diabetic status in relation to critical organ weights and blood lipids, *random *or *fasting *blood glucose were compared to liver and kidney weights, as well as fasting plasma triglycerides and total cholesterol at the end of the study (Figure. 4). The strongest correlation existed between either *fasting *(r = 0.91) (data not shown), or *random *(r = 0.84) blood glucose and liver weight, and to a lesser extent with kidney weight (r = 0.64), but total adipose mass was unrelated to blood glucose (Figure. 4A,B,C). Total fasting plasma cholesterol (r = 0.77) and triglycerides (r = 0.74) were equally well-correlated with *random *blood glucose (Figure. 4D,E), which was slightly better than correlations with *fasting *blood glucose (data not shown).

### Body fat and diabetes onset (Expt 4)

Results in Table 2 further explore the relationships between diabetes onset, organ weights (including fat pads), and plasma lipids in a long-term experiment. The first 10 rats killed at 7 mo divided equally into diabetic (n = 5) or nondiabetic (n = 5) groups based on random blood glucose >150 mg/dl, similar to the pattern seen in Expt 2. Although food and water intake were not measured in the 7 mo rats, body weight was significantly greater in the 5 rats that had developed diabetes. After 16 h fasting, blood glucose was 50% higher in diabetics, but was not significant due to variation. Their livers were significantly enlarged at necropsy, while kidneys tended to be larger. Total adipose accumulation was similar between diabetics and nondiabetics. Both total cholesterol and triglycerides tended to be increased in this early-onset group (Table S1, Additional file 1, 7 mo).

**Table 2:** Body and organ weights, random blood glucose and plasma lipids for 7 and 11 mo old male Nile rats fed chow (#5020) with different onset to diabetes (Expt 4)

	7 mo		11 mo	
	
	Non-diabetic	Diabetic	Non-diabetic	Diabetic
	R. blood gluc <150 mg/dL	R. blood gluc >150 mg/dL	R. blood gluc <150 mg/dL	R. blood gluc >150 mg/dL
	
	n = 5	n = 5	n = 3	n = 7
Body weight, terminal (g)	112 ± 7	127 ± 13*	126 ± 10	124 ± 13
				
Food intake last mo (kcal/d)	NA	NA	37 ± 3	76 ± 15*
				
Water intake last mo (mL/d)	NA	NA	10 ± 2	72+8*
				
Blood glucose (mg/dL)				
Random	75 ± 43	266 ± 81*	98 ± 43	448 ± 39*†
15 h fasting	43 ± 15	67 ± 25	79 ± 64	210 ± 99†
				
Organ weight (%BW)				
Liver	2.85 ± 0.32	4.35 ± 1.02*	2.66 ± 0.34	6.70 ± 0.62*†
Kidney	0.77 ± 0.15	1.00 ± 0.21	0.82 ± 0.13	1.37 ± 0.26*
Adipose				
Perirenal	1.66 ± 0.66	2.28 ± 0.53	2.00 ± 0.91	0.68 ± 0.22*†
Epididymal	4.12 ± 0.71	2.88 ± 0.74	3.23 ± 0.70	2.53 ± 1.01
Suprascapular (brown fat)	1.90 ± 0.48	2.02 ± 0.37	1.97 ± 0.20	1.44 ± 0.73
Total fat (all above)	7.68 ± 1.31	7.18 ± 1.31	7.20 ± 1.61	4.64 ± 1.36†
				
Plasma				
TC (mg/dL)	141 ± 21	173 ± 43	122 ± 26	391 ± 140*†
TG (mg/dL)	49 ± 16	124 ± 86	70 ± 16	659 ± 626

Values are mean ± SD, total n = 20, NA = not available

* Significant difference between non-diabetic and diabetic rats at 7 mo. (p < 0.05)

† Significant difference between 7 and 11 mo, (p < 0.05)

By contrast, only 3 of the 10 rats killed after 11 mo were still nondiabetic based on random and fasting glucose, whereas 7 were diabetic. Body weights were now similar, but liver and kidneys in the diabetic rats were double the size of those in nondiabetics; and now the perirenal adipose pool was significantly reduced in diabetics, while blood lipids were extremely elevated, especially triglycerides in individual rats (Table 2).

#### Insulin resistance and early progression (Expt 5)

Table 3 details the relationships between body weight, fasting insulin, and plasma lipids, expressed in terms of diabetes stages that developed after different ages at weaning (separation) age and progressively longer exposure to diet challenge. For this experiment, 36 male Nile rats were weaned when 4-5 wks old and followed for 2 wks; a second group of 27 males were separated at 6-7 wks and followed for 4 wks; while a third group of 20 was separated at 8-9 wks and followed 12 wks. All were housed individually and fed the high-fat, low-fiber Western-type diet (Table 1). The question was how early and what degree of diabetes could be detected following a challenge diet? Within each of the three age groups, four stages of diabetes were identified (Table 3). Stage A represented low insulin (<3.5 ng/ml) and low random blood glucose (<150 mg/dl). Development of insulin resistance (stage B) was defined by elevated insulin (>3.5 ng/ml) and low random blood glucose (<150 mg/dl). Stage C (entrenched diabetes) was characterized by both elevated insulin (>3.5 ng/ml) and random blood glucose (>150 mg/dl); Stage D represented depressed insulin, while blood glucose and lipids were still elevated (beta-cell failure). The latter was only achieved in the oldest rats (followed for the longest time).

**Table 3:** Crossectional comparison in male Nile rats separated between 4 and 9 wks and fed high-fat, low-fiber Western-typediet for 2 to 12 wks to assess stages of insulin resistance (Expt 5)

	Stages			
	
	A	B	C	D
Age at separation				
4-5 wks (followed for 2 wks)				
BW (g)	70 ± 11^a,b^	79 ± 9^a^	83 ± 13^b^	
n = 36	13 (36%)	18 (50%)	5 (14%)	0
Insulin (ng/mL)	1.0 ± 0.8^a,b^	7.7 ± 3.1^a^	6.3 ± 1.7^b^	
Random blood glucose (mg/dL)	72 ± 33^a^	77 ± 33^b^	223 ± 65^a,b^	
TG (mg/dL)	53 ± 23^a^	69 ± 21^b^	145 ± 75^a,b^	
TC (mg/dL)	91 ± 33	96 ± 19	105 ± 33	
				
Age at separation				
6-7 wks (followed for 4 wks)				
BW (g)	89 ± 10^a,b^	101 ± 11^a^	103 ± 12^b^	
n = 27	11 (41%)	11 (41%)	5 (19%)	0
Insulin (ng/mL)	1.9 ± 1.1^a^	6.8 ± 2.2^a^	5.4 ± 3.8	
Random blood glucose (mg/dL)	44 ± 12^a^	59 ± 25^b^	341 ± 154^a,b^	
TG (mg/dL)	37 ± 10^a^	50 ± 21^b^	286 ± 129^a,b^	
TC (mg/dL)	57 ± 11^a^	80 ± 15^b^	117 ± 72^a,b^	
				
Age at separation				
8-9 wks (followed for 12 wks)				
BW (g)	101 ± 14^a,b^	120 ± 7	126 ± 14^a^	133 ± 23^b^
n = 20	3 (15%)	6 (30%)	8 (40%)	3 (15%)
Insulin (ng/mL)	1.4 ± 0.4^a,b^	7.4 ± 2.1^a,c^	9.0 ± 4.3^b,d^	1.7 ± 0.9^c,d^
Random blood glucose (mg/dL)	62 ± 17^a,b^	70 ± 6^c,d^	340 ± 165^a,c,e^	534 ± 72^b,d,e^
TG (mg/dL)	55 ± 16^a^	66 ± 7^b^	192 ± 141^a,b^	132 ± 26
TC (mg/dL)	129 ± 16	111 ± 19	172 ± 108	176 ± 25

Values are mean ± SD

a,b,c,d,e Means in row sharing a common superscript are significantly different (P < 0.05) using one-way ANOVA and Fischer's PLSD test.

Stage A = normal, Stage B = elvevated insulin, Stage C = elevated insulin and glucose, Stage D = declining insulin, elevated glucose

Already by 6-10 wk of age, and 2-4 wks of challenge diet, more than 60% of these male rats had progressed beyond Stage A. By 20 wks of age and 12 wks of diet, 85% had moved beyond Stage A. Between 7 wks and 20 wks of age the number of rats in Stage C had essentially tripled and a small percentage (15%) now showed in Stage D. Insulin typically increased about 7-fold by Stage B (in approximately 50% of the younger rats), and peaked slightly above this level at 16-20 wks, before declining into Stage D. The rise in blood glucose, a delayed rise in TG, paralleled insulin dynamics, except glucose continued to increase when insulin declined in Stage D. An increase in TC was less dramatic. In general, for all three age groups, rats that had progressed to stages B and C, (or stage D for the oldest rats), were those that gained significantly more weight than those still in stage A, ie. they grew at a faster rate.

### Early vs. late diabetes in young rats (Expt 6)

Based on the trend in experiment 5, we determined whether young rats would rapidly separate into early-onset and late-onset diabetes based on growth efficiency and calorie intake. Accordingly, 8 male Nile rats were weaned at 5 wks and fed the high-fat, low-fiber Western-type diet for 4 wks. When sacrificed, they were categorized as early- or late-onset diabetes based on their weekly random blood glucose history (Table 4). Similar to rats weaned and killed at older ages (Expt 3 and 4), the early-onset group had gained significantly more weight (14%) than late-onset rats after only 2 wks on diet, which was still apparent after 4 wks when sacrificed. Interestingly the early-onset rats revealed greater skeletal length (6%), relatively less carcass mass, more adipose than the late-onset rats. This was related to greater food and water intake measured during the first 2 wks in the early-onset group, but was more strikingly linked to the 2 wk growth efficiency (18 kcal/g BW gain) which was significantly better (33%) than the late-onset group during that interval (28 kcal/g BW gain). The terminal random blood glucose after 4 wks also differed at 344 mg/dl compared to 46 mg/dl, respectively. Unlike longer experiments, neither liver nor kidneys were enlarged, but triglycerides, insulin and HbA1c were significantly elevated in the diabetic rats (Table 4). However, neither BMI nor LMI were affected by diabetic status, suggesting that neither the relative overall fat mass nor carcass mass (muscle/bone) were appreciably altered from a total body perspective.

**Table 4:** Body and organ weights, blood glucose, fasting insulin and plasma lipids for 5-wk old male Nile rats with early- or late-onset diabetes when fed a high-fat, low-fiber purified diet for 4 wks (Expt 6)

	High-fat, low-fiber diet (Western-type)	
	
	Early-onset	Late-onset
Body weight (g)		
Initial (age: 5 wks)	62 ± 6	61 ± 4
After 2 wks	90 ± 6	79 ± 7*
After 4 wks	104 ± 6	90 ± 8*
		
Body weight gain (wks 5-7) (g/d)	2.2 ± 0.1	1.3 ± 0.4*
		
Food Intake (wks 5-7) (g/d)	14.8 ± 0.6	12.3 ± 0.9*
(kcal/d)	40.5 ± 1.7	33.8 ± 2.8*
Growth efficiency (kcal/d/g BW gain)	18.5 ± 1.1	28.2 ± 7.1*
		
Water Intake (wks 5-7) (mL/d)	15 ± 6	9 ± 5
Random blood glucose (mg/dL)		
Initial (age: 5 wks)	110 ± 55	79 ± 18
After 4 wk	344 ± 80	46 ± 10*
Organ weight (%BW)		
Liver	3.87 ± 0.84	3.22 ± 0.42
Kidney	0.73 ± 0.11	0.70 ± 0.13
Adipose		
Perirenal^¥^	2.65 ± 0.33	1.82 ± 0.76*
Epididymal	5.40 ± 0.57	3.80 ± 0.98*
Omental	2.61 ± 0.38	2.05 ± 0.59
Inguinal	1.64 ± 0.20	1.39 ± 0.42
Suprascapular (brown fat)	2.18 ± 0.16	1.52 ± 0.49
Total fat (all above)	14.5 ± 1.5	10.6 ± 2.8*
Carcass	67.2 ± 0.9	72.8 ± 2.2*
Body length (cm)	13.4 ± 0.2	12.6 ± 0.5*
BMI (kg/m^2^)	5.8 ± 0.4	5.5 ± 0.2
LMI (Carcass wt kg/m^2^)	3.9 ± 0.2	4.0 ± 0.1
Plasma		
TC (mg/dL)	88 ± 26	63 ± 4
TG (mg/dL)	126 ± 14	96 ± 19*
Insulin (ng/mL)	18.2 ± 3.4	5.3 ± 3.2*
HbA1c (%)	6.5 ± 0.7	5.3 ± 0.4*

Values are mean ± SD (n = 4)

* Significantly different (P < 0.05)

¥ Represents perirenal + retroperitoneal fat pad.

### Glucose response to high-fat versus low-fat diets (Expt 7)

In order to explore the effect of dietary fat and fiber on Nile rat diabetes, purified diets were compared to chow in twelve 5 wk-old male rats (Table 5). The high-fat, low-fiber diet (Western-type diet, 4.5 kcal/g), described above was compared to a low-fat, high-fiber Mediterranean-type diet, 3.5 kcal/g (Table 1) along with a third group fed chow as control at 3.57 kcal/g. Although the high-fat, low-fiber group consumed 20% less energy per day compared to the low-fat, high-fiber group (n.s.) the weight gain/d in the former was 10% greater after 20 wks (n.s.). However, the total adipose pool did not differ between the three groups at study end, and the cecum was largest (n.s.) in the low-fat, high-fiber group. Initially, at 5 wks the random blood glucose was similar for all groups analyzed, but after 24 wks the low-fat group had the lowest fasting blood glucose, even though its random blood glucose was highest (Table 5).

**Table 5:** Food intake, body and organ weights, blood glucose and plasma lipids for 5-wk old male Nile rats fed 3 types of diets for 24 wks (Expt 7)

	Diet:		
	
	#1	#2 (Western-type)	Chow
	Low-fat, high-fiber	High-fat, low-fiber	(RMH #3000)
**CHO: FAT: Prot %en**	**69:15:16**	**40:43:17**	**60:14:26**

Body weight (g)			
Initial (5 wks old)	51 ± 3	52 ± 7	55 ± 5
Final (24 wk trial)	110 ± 11	118 ± 19	131 ± 9
			
Body weight gain (g/d)	0.34 ± 0.06	0.38 ± 0.09	0.41 ± 0.07
			
Food intake as dry diet (g/d)	22.5 ± 1.5	14.3 ± 1.0**†**	N/A
(kcal/d)	79 ± 5	64 ± 4	N/A
			
			
Organ weight (%BW)			
Liver	5.39 ± 0.54^a^	7.23 ± 1.06^a,b^	4.48 ± 1.41b
			
Kidney	1.26 ± 0.31	1.47 ± 0.33^a^	0.98 ± 9.30^a^
			
Cecum	3.3 ± 0.9	2.1 ± 1.0	2.1 ± 1.3
			
Adipose			
Perirenal*	0.80 ± 0.59	0.73 ± 0.62	1.34 ± 0.53
Epididymal	2.25 ± 0.69^a^	2.59 ± 0.82	3.33 ± 0.35^a^
Inguinal	0.73 ± 0.42	0.72 ± 0.25	0.85 ± 0.15
Total (all above)	3.79 ± 1.62	4.04 ± 1.64	5.52 ± 0.98
			
Carcass	68 ± 3	68 ± 3	69 ± 4
Initial blood glucose (mg/dl)	61 ± 4	81 ± 13	N/A
Random, 24 wk glucose (mg/dl)	395 ± 149^a^	276 ± 82	150 ± 82^a^
Fasting, 24 wk glucose^Á ^(mg/dl)	76 ± 17	158 ± 114	118 ± 142
			
Liver Lipids			
TC (mg/g)	3 ± 1^a^	15 ± 3^a,b^	5 ± 2^b^
TG (mg/g)	18 ± 8^a^	137 ± 40^a,b^	35 ± 6^b^
			
Plasma (fasting, terminal^Á^)			
TC (mg/dL)	199 ± 51	568 ± 400^a^	97 ± 14^a^
TG (mg/dL)	194 ± 26^a^	1371 ± 992^a,b^	203 ± 78^b^

Values are Mean± SD (n = 4). NA = not available.

^a,b,c.. ^Means in a row sharing a common superscript differ (p < 0.05) using one-way ANOVA and Fisher's PLSD test.

^Á^Blood glucose after 16 h fasting. **† **Significantly different (p < 0.05).

*Perirenal represents the perirenal and retroperitoneal fat pool

**Figure 5:**
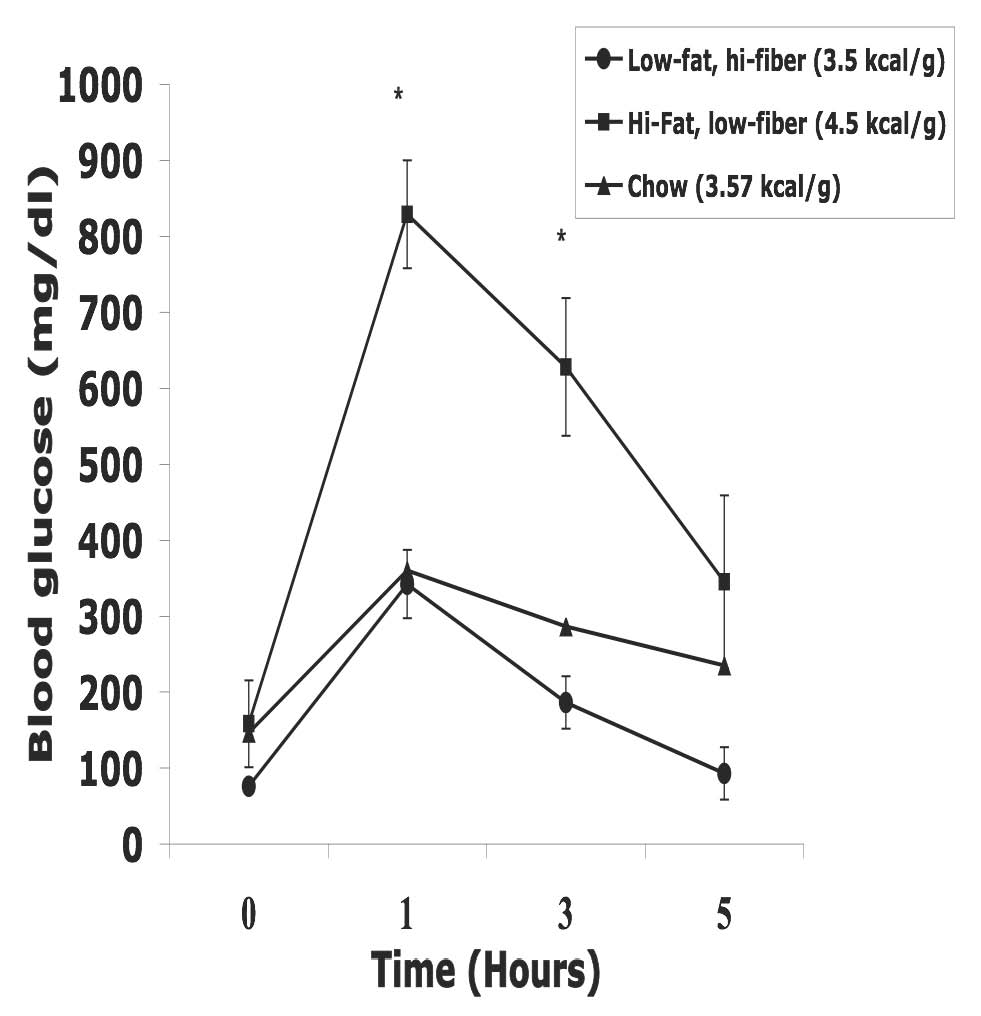
**(Expt 7)**. Intraperitoneal Glucose Tolerance Test for male Nile rats (n = 4) fed chow and two semi-purified diets, either low-fat, high-fiber; high-fat, low-fiber; or chow (#3000) diets for 20 wks. The figure depicts blood glucose change over time (mean ± SEM) post glucose injection (2.5 g/kg BW). * Significantly different (p < 0.05)

The ipGTT after 20 wks on diet (Figure. 5) indicates that rats fed the high-fat, low-fiber diet had an elevated baseline blood glucose (146 mg/dl) that peaked at 829 mg/dl after one hour. After 5 h it was still 345 mg/dl. The chow group averaged the same blood glucose at baseline (at 146 mg/dl), but only rose to 360 mg/dl by one hour and 235 mg/dl after 5 h, still well above the initial value. By contrast, the low-fat, high-fiber group had the lowest 0-time blood glucose at 76 mg/dl, which reached 342 mg/dl at 1 h and returned to 96 mg/dl after 5 h.

After 24 wks, liver and kidney weights, liver TC, and TG, as well as plasma TG were significantly greater in rats fed the high-fat, low-fiber diet compared to chow (Table 5).

### Calorie restriction studies (Expt 8)

Nile rats were fed either rat chow (#5020) ad libitum or that diet restricted to 75% of ad libitum for 18 wks (Table 6). When ipGTT was assessed, the calorie-restricted rats had an initial fasting blood glucose of 72 mg/dl, compared to 211 mg/dl for rats fed ad libitum. At 1 h the ad libitum group rose to 517 mg/dl, more than twice that of the restricted group at 226 mg/dl. The only significant difference in organ weights occurred in the liver, which was larger by 20% (p < 0.05) in the ad libitum diabetic rats compared to the restricted group. Even though body weight did not differ between groups, the *fasting *blood glucose was significantly lower in the restricted group after 13 wks (36 mg/dl versus 70 mg/dl), as well as terminally (72 mg/dl versus 211 mg/dl) after 18 wks.

**Table 6:** Food and water intake, body and organ weights, blood glucose and plasma lipids for 20-wk old Nile rats fed chow #5020 ad libitum or restricted to 75% ad libitum for 18 wks (Expt 8)

	Chow 5020	
	
	ad libitum	Restricted to 75%
**CHO:FAT:Prot %en**	**57:21:22**	**57:21:22**
**Diet: Kcal/g**	**3.75**	**3.75**

Body weight (g)		
Initial (age: 20 wks)	112 ± 20	102 ± 8
Final after 13 wks	119 ± 23	110 ± 9
Final after 18 wks	123 ± 21	112 ± 15
gain/day	0.019 ± 0.007	0.020 ± 0.024
		
Food intake (g/d)	17.4 ± 6.8	13.0 ± 3.0
(kcal/d)	65 ± 25	49 ± 11
		
Water intake (ml/d)	19.9 ± 15.0	20.7 ± 14.4
		
Organ weight (%BW)		
Liver	4.36 ± 0.98	3.40 ± 0.36*
Kidneys	1.01 ± 0.24	0.89 ± 0.17
Cecum	1.86 ± 0.5	1.61 ± 0.28
Adipose		
Perirenal	1.21 ± 0.54	0.91 ± 0.70
Epididymal	2.26 ± 1.06	2.35 ± 0.70
Inguinal	1.45 ± 0.39	1.15 ± 0.23
Omental	1.09 ± 0.16	0.88 ± 0.29
Suprascapular (brown fat)	3.01 ± 1.18	1.80 ± 0.74
Total (all above)	9.03 ± 2.21	7.10 ± 2.05
Carcass	68.5 ± 2.86	70.0 ± 3.71
		
Body Length	13.7 ± 0.9	13.5 ± 0.4
BMI (kg/m^2^)	6.5 ± 0.3	6.2 ± 0.5
LMI (kg/m^2^)	4.4 ± 0.3	4.3 ± 0.3
		
ipGTT at 18 wk		
0 time	211 ± 152	72 ± 35*
1 hr	517 ± 172	226 ± 135*
		
Blood glucose (15 h fasting), mg/dL		
Initial	56 ± 19	60 ± 41
After 13 wks	70 ± 21	36 ± 3*
Final after 18 wks	211 ± 152	72 ± 35*
Plasma lipids (terminal)		
TC (mg/dL)	169 ± 68	115 ± 22
TG (mg/dL)	179 ± 149	56 ± 19

Values are Mean ± SD, (n = 5-6; 2-3 females and 2-4 males per group)

* Significanly different (p < 0.05)

### Blood pressure (Expt 9)

In order to follow blood pressure, 13 male Nile rats were weaned to chow (Lab Diet, #5020) at the age of 8 wks and assessed for blood pressure over 21 weeks, starting at 5 mo of age, ie. when these rats distributed into early-onset or late-onset groups based on fasting blood glucose. Glucose became elevated by 22 wks of age in 3 rats (early-onset), while the remaining 10 began an equivalent rise only when about 40 wks old. The blood pressure rise lagged the blood glucose profile somewhat, becoming elevated in the early-onset group when 34 wk old and increasing or remaining elevated thereafter. BP increased in late-onset rats at 43 wks and then approximated the early-onset group. Urinalysis conducted in the early-onset rats revealed polydipsia and polyuria with typical signs of kidney failure, including microalbuminuria and hyperglucosuria, sometimes even ketones (data not shown). No difference in body weight was observed over the comparison period, even though the early-onset group started to loose weight slightly after 27 wks of age, indicating late-stage diabetes.

Marked elevations in fasting glusose and plasma lipids in the early-onset group at study end indicated that their diabetes was, indeed, more advanced, and that they had proceeded to stage D where the plasma insulin was depleted as result of beta-cell failure (Table 7).

**Table 7:** Blood glucose and blood pressure dynamics for 13 male Nile rats fed chow (#5020) divided at 22 wks into early-onset and late-onset groups followed for 21 wks (Expt 9)

	Groups	
	
	Early-onset	Late-onset
Fasting blood glucose (mg/dL)		
22 wk of age	271 ± 34	77 ± 34*
27 wk of age	406 ± 73	137 ± 92*
34 wk of age	371 ± 124	71 ± 20*
40 wk of age	339 ± 43	211 ± 120*
43 wk of age	425 ± 33	288 ± 196*
		
Systolic blood pressure (mmHg)		
27 wk of age	128 ± 8	123 ± 10
34 wk of age	156 ± 8	134 ± 6*
40 wk of age	171 ± 13	137 ± 7*
43 wk of age	164 ± 6	158 ± 7
		
Body weight (g)		
Initial (age: 8 wks)	83 ± 5	76 ± 4
22 wk of age	115 ± 13	117 ± 12
27 wk of age	116 ± 12	125 ± 10
34 wk of age	113 ± 18	127 ± 11
40 wk of age	114 ± 14	125 ± 10
43 wk of age	109 ± 12	123 ± 11
		
Plasma (43 wk of age)		
Insulin (ng/mL)	0.9 ± 0.9	3.0 ± 2.0*
TC (mg/dL)	413 ± 302	139 ± 98*
TG (mg/dL)	1472 ± 1282	253 ± 245*

Values are mean ± SD (early-onset n = 3, late-onset n = 10)

* Significantly different (P < 0.05)

## Discussion

This report introduces the Nile rat as a novel model of Metabolic Syndrome associated with diet-induced diabetes in a readily managed lab rodent of intermediate size. The diabetes was age, sex, and diet dependent, and appeared related to stressful gene-environment interactions that affected energy utilization. It is unique in its high percentage of involvement (close to 100% in males, somewhat less in females), and in the fact that several aspects of the disease examined in this report (early abdominal obesity, hyperinsulinemia, elevated blood glucose and triglyceride plus hypertension, as well as unpublished data on depressed HDL) mimic Metabolic Syndrome and type 2 diabetes in humans [9,12-15].

### Diet and Growth considerations

Several aspects of diabetes in Nile rats are noteworthy from a nutritional point of view, particularly the disposition of energy for growth or storage as adipose. Specifically, males fed a chow diet (Lab Diet, #5020, 3.57 kcal/g) grew faster than females (as expected), and they developed diabetes more readily than females, presumably based on sex hormone differences. Even among individual males, those that grew faster, reminiscent of human infants with a rapid postnatal weight gain [16-19], developed diabetes sooner (early-onset groups).

One might assume that more rapid weight gain represented greater food consumption leading to adipose accumulation and diabetes. However, only for a brief 2 wk period in the youngest weanlings, age 5 to 7 wks (Expt 6), could we detect early-onset diabetes linked to greater food intake (20%) and greater adiposity (37%). Thus, linear growth was faster (body length), relative muscle mass less (carcass % bd wt) and fat depots greater (adipose % bd wt) for early-onset rats. But even there, greater growth efficiency (33% better) had a greater impact than the increased calorie intake. In a second experiment where food intake was tracked for 32 wks beginning at 8 wks of age (Expt 3), calories consumed did not differ as a function of diabetes onset, but better growth *efficiency *(100%) still pertained for the early-onset group. Expt 3 also teaches that growth efficiency, as a causal link for diabetes onset, only applied during rapid growth, as the relationship between calorie intake and body weight gain disappears once growth ceases. When growth is complete and diabetes established, calorie intake and efficiency measures of energy utilization for growth would no longer pertain.

In late stages of the disease in older rats (Table 2, 11 mo), blood glucose and plasma lipids were severely elevated, and the rats developed insulin-dependent diabetes with adipose wasting, especially in the perirenal fat pad, even as calorie intake doubled. This combination of wasting adipose plus elevated plasma triglycerides in advancing diabetes, mimics the defect of free fatty acid recycling seen in hyperinsulinemic humans with insulin resistance and diabetes [9], confirming the comparable metabolic profile in these Nile rats.

Thus, rats that used calories more efficiently to (atypically) accelerate linear growth and early weight gain, including adipose accumulation relative to muscle mass, were more prone to insulin resistance and diabetes later on. In addition, a shift to fat catabolism (especially perirenal fat) in the later stages of diabetes may have complicated our estimates of adiposity in cross-sectional studies, since adipose tissue may increase initially then begin to decline, depending on the rat age and stage of diabetes. It is important to note that, in general, the diabetes did not appear to depend on hyperphagia or generalized obesity *per se*, as total body fat never exceeded 10 to 15% of body weight and, in most experiments, did not correlate with blood glucose. This differs substantially from most other mouse or rat models of diabetes.

#### Wild Nile rats

The relationship between rapid growth and diabetes is supported by the observation that Nile rats in the wild, where food and calories are less available, grow less rapidly than captive rats and do not develop diabetes. In fact, body weight of wild Nile rats, even in the rainy season when food is more abundant, was about 35% less in males (ie. 85 g final wt) and 40% less in females (65 g final wt) than comparable weights of captive-fed rats [20]. During the dry season both male and female wild rats weighed about 60% less than captive-fed rats. Nonetheless, wild rats reach their reduced adult weight in approximately the same amount of time (5 mo) as captive rats. These differences also reflect the fact that their grasses-and-insects diet in the wild has a caloric density close to 2.0 kcal/g [1], compared to the 3.2-4.5 kcal/g in our lab diets. Thus, if food is curtailed by natural environmental conditions linked to the dry season [20] or by food restriction in the laboratory setting, diabetes risk is reduced.

To this point, two of the present experiments demonstrate that dietary factors influenced the onset and degree of diabetes observed, similar to the situation for type 2 diabetes in humans [21] and sand rats [3,5,22,23]. On the one hand, reducing calorie intake to 75% ad libitum affected weight gain only slightly (n.s.), but limited diabetes, protecting against fatal disease much as modest weight loss does in humans with type 2 diabetes [24]. Sand rats reportedly even reverse their diabetes if food intake is restricted 50% before beta-cell failure occurs [3,25]. In the second instance a high-energy, Western-type diet (4.5 kcal/g), which would potentiate fatty acid oxidation and fat storage, significantly elevated blood glucose and rendered Nile rats severely glucose-intolerant relative to a low-fat, high-fiber Mediterrean-type diet (3.5 kcal/g). Interestingly, despite these differences in dietary energy density and composition, rats compensated with the result that their total energy intake and body fat pools did not differ for the 24 wk comparison. Furthermore, even though the *random *blood glucose at study end was highest for the low-fat (high-CHO) group, that group also had the lowest *fasting *glucose (Table 5). This suggests that the low-fat diet still allowed for reserve insulin secretion to reduce an elevated random glucose during the 16 h fast. Thus, the rate and composition of energy processed by the Nile rat influences type 2 diabetes onset without necessarily revealing outward signs on body weight and adiposity. From these observations it is not surprising that food restriction and weight loss are the first method of choice for alleviating diabetes in humans afflicted with type 2 diabetes [24].

### "Thrifty genes" and insulin resistance

The response to restricted calorie intake and enhanced calorie utilization for growth in Nile rats with early-onset diabetes is also reminiscent of the sand rat, where evidence for the "thrifty genes" theory was presented to explain the prevalence of diabetes in a susceptible strain, compared to a more resistant one [3,12,26,27]. In that situation diabetes-prone sand rats experienced 33% greater feed efficiency and became insulin resistant, eventually storing more energy as body fat per calorie consumed than the resistant strain. Unfortunately, no data were presented for carcass or muscle mass representing somatic growth rate. In the same manner, both older and younger cohorts of Nile rats with early-onset diabetes (Expts 3 and 6) were more energy efficient than the late-onset groups of the same age; and those in Expt 6 also accumulated more fat early, indicating that the early-onset group (and presumably expression of their Metabolic Syndrome) was related to genetic control of energy utilization, particularly in young rats.

These data suggest that genetically controlled *growth rate *is modulated by diet composition, including dietary calorie density [3] and availability, possibly including the specific macronutrient composition of the diet itself. For example, high-fat, low-fiber diets are notorious for inducing diabetes in susceptible models [22]. Once growth ceased and energy shifted from linear skeletal growth and muscle expansion to maintenance/storage, it would appear that insulin resistance (initiated during rapid growth) led to sustained hyperglycemia [28]. Our late-onset rats simply grew slower, so they delayed their destiny with diabetes because they required more calories for less growth (less efficient) early on, presumably reducing calories available for storage. In sand rats, which are ecologically and physiologically similar to Nile rats [4,23,25], insulin resistance precedes hyperglycemia, similar to humans and Nile rats [29]. One current theory suggests that excessive fatty acid oxidation in muscle mitochondria leads to ROS-induced damage that initiates insulin resistance as a means to protect muscle from the burden of additional energy disposal [29,30]. Another theory suggests that excessive uptake of free fatty acids by tissues, including inflammation and death of pancreatic beta-cells, leads to insulin resistance and fat accumulation in liver and muscle [31]. To date the role of inflammation has not been explored as a factor contributing to the Metabolic Syndrome of Nile rats.

Similar to early "growth stress" in Nile rats, epidemiological studies have noted that small-for-term infants growing faster at 7 years of age than infants with normal birth weight were more likely to develop type 2 diabetes as adults [32]. It is thought that *in utero *nutrition is a key dynamic [33,34], but nothing is known about the specifics of the nutrients involved or the character of postnatal nutrition, other than high energy intake being a risk factor affecting the incidence and onset of their diabetes [35]. It will be important to define the maternal metabolism and nutrient intake of the Nile rat during pregnancy, as well as details relative to postnatal diet composition and behaviors around food intake that impact the growth rate of their pups, particularly males.

In any event the Nile rat model, with a focus on growth and food (energy) utilization during rapid growth between 3-10 wks of age, provides an opportunity to identify diet-gene interactions underlying insulin resistance and diabetes. With this paradigm, it should be possible to intervene with diet or drug to establish relative efficacies for prevention of overt, diet-induced insulin resistance and diabetes in experiments of 4-7 weeks, which is unique among animal models fully expressing this disease from natural causes.

### Model comparisons

Animal models are useful for studying the pathophysiology of human disease and allow for therapeutic intervention with an abbreviated time span relative to human experiments. The model should mimic the human disease, or at least present its major symptoms for study. So far no animal model mirrors all characteristics of the Metabolic Syndrome and non-insulin-dependent (type 2) diabetes mellitus [36]. Many mouse and rat models have idiosyncratic similarities to certain aspects of the human condition, often attributed to a specific gene mutation. For example, the ob/ob [obese], db/db [diabetes] and the Zucker diabetic fatty rat (ZDF) have mutations either in the leptin gene (ob/ob) or in the leptin receptor (db/db) which causes over-eating obesity, unlike the natural history of the disease in humans where leptin is seldom implicated [37,38]. Gene analysis through backcross breeding of diabetes-resistant and diabetes-prone sand rats suggests that a single major gene may control the transition from normo- to hyperglycaemia in that model [39]. Still another sand rat report labeled larger male rats as "obese" (with <6% of total body weight as fat), and suggested their "obesity" was the reason for their insulin resistance and diabetes. However, differential growth measures distinguishing between lean body growth (lean mass *per se*) and BMI (adiposity) were lacking, so the question remains whether these desert rodents develop diabetes due to accelerated growth rates or overabundant fat accumulation, or both [6]. The C57BL/6 mouse is more susceptible to diet-induced obesity (DIO) and diabetes than other mouse strains [40], but its diabetes is not as progressive or severe as that in either the Nile rat or sand rat, and does not present all sequelae present in the human disease. This is true even though the DIO mice often double their weight from added adipose. Plus, clinical aspects of diabetes in DIO mice are subtle and of minimal clinical consequence, taking 6-12 mo to develop. Extreme overall obesity is not characteristic of diabetes in Nile rats or sand rats, evidenced by the 10 to 15% upper limit in body fat observed herein, and the 2-6% range in male or female sand rats [6].

The utility of the Nile rat model should be emphasized from several aspects. First, it appears to be a polygenic wild-type rodent (outbred) that expresses all aspects of the Metabolic Syndrome measured to date, including abdominal fat accumulation (at least in young rats), hyperinsulinemia, hyperglycemia, hypertriglyceridemia (with depressed HDL, unpublished data), microalbuminuria (data not shown), and hypertension on the way to terminal type 2 diabetes. Second, as in the sand rat [6], the diabetes has 5 clearly marked stages leading to ketosis and death, which are analogous to human type 2 diabetes. Pathology of the liver, kidney, and pancreatic beta cells appear to be similar to human disease [41] and invite investigation of issues still outstanding around type 2 diabetes in humans, eg. mechanisms and interventions to improve insulin secretion or kidney function associated with normal progression of the diet-induced disease. Third, the incidence of diabetes is high in this captive-bred model, slightly greater than that reported for out-bred sand rats [6]. Incidence in the sand rat has been enhanced further by selective inbreeding in an Israeli colony [3]. Almost all captive male Nile rats fed chow become diabetic by 1 year, some as early as 6-10 wks. In weanling sand rats diabetes developed in a susceptible inbred strain within 2 wks on a challenge diet (only 2.9 kcal/g vs. our Nile rat challenges of 3.57 kcal/g in chow or 3.5 kcal/g and 4.5 kcal/g in our purified diets) followed by ketosis and death in 10 wks [3]. By contrast, to date in our older Nile rat cohorts, even our higher density diet produced a less acute, more prolonged, but all-inclusive disease in males. Female Nile rats delay diabetes onset, with approximately 75% eventually developing the disease by 1-1.5 yr. Similarly, severity and incidence of diabetes in captive female sand rats was slightly less than males at 16 wk of age when fed a chow diet in an outbred colony [6]. Many data suggest that type 2 diabetes may be more common in men than women, at least premenopausal women [32,42,43].

Certain physiological correlates of the diabetes in Nile rats are noteworthy. First, blood glucose was highly correlated with hepatomegaly, which reflected hepatic steatosis similar to humans with type 2 diabetes [44]. Second, kidney enlargement with polyuria, and eventual renal failure ending in glomerular and interstitial nephritis and sclerosis with reduced kidney size were associated with polydypsia, polyuria, and ketosis at the endstage of the disease linked to elevated glucose (unpublished data). Diabetes progression also was associated with elevated blood pressure, which is a frequent morbid association in humans with diabetes [45,46]. These observations, coupled with the hyperlipemia that develops as glucose rises, suggests that abnormal energy metabolism based on liver handling of insulin and calories leading to insulin resistance are critical aspects of the disease in Nile rats, even as they are in humans with the type 2 diabetes and Metabolic Syndrome [47].

### Random versus fasting glucose

One practical issue examined was whether *random *or *fasting *glucose is the better measure of this diabetes. While both measures demonstrate disease progress, the random value is easier to execute, as it does not require prolonged fasting. Because Nile rats are diurnal, they eat predominantly during the day, so a random glucose value is never far removed from eating, which can be considered a continuous stimulus for insulin secretion during daytime [2]. Furthermore, first signs of glucose intolerance (insulin resistance) can be detected easier with excursions in random blood glucose than with fasting glucose. A 16 h fasting glucose value, on the other hand, provides the opportunity to determine whether enough insulin can still be generated to clear circulating glucose, thereby demonstrating the reserve capacity of beta-cell function in the host. When insulin secretion is waning in Nile rats, fasting glucose remains elevated, even as it does with fasting in advanced type 2 diabetes in humans [14,24,48].

Thus, in this model energy (glucose, triglycerides) is not removed from systemic circulation effectively despite elevated insulin (hyperinsulinemia reflecting insulin resistance), which leads to a blood glucose increase that eventually leads to beta-cell failure with decreased insulin production [47,49,50]. The cutoff points for blood glucose differ for fasting or random glucose, but they were highly related. Random glucose above 150 mg/dl was a good predictor of diabetes from the current data, while fasting glucose >110 mg/dl seems reasonable for diabetes onset. *Random *glucose commonly rises to 300-500 mg/dl, often over 600 mg/dl, in Nile rats with advanced diabetes and insulin depletion, whereas *fasting *glucose seldom exceeds 400 mg/dl, even in the worst case of diabetes. Slower progressing diabetes in older Nile rats also was associated with a much greater hyperglycemia and hyperlipemia, with elevated VLDL-TG (up to10-fold), total cholesterol (5-fold) and HDL depressed to 1/5 normal (unpublished data). By contrast, triglycerides and cholesterol reportedly rise only minimally in sand rats [51].

## Conclusion

The data presented indicate that the African grass rat (Nile rat) represents a novel model of Metabolic Syndrome associated with diet-induced type 2 diabetes, expressing developmental growth and nutritional correlates similar to those in humans. All aspects of the Metabolic Syndrome and the Insulin Resistance Syndrome examined to date in captive Nile rats appear to mimic those of type 2 diabetes in humans [9,14,48,52]. Like humans, males appear more susceptible than females, with insulin resistance and diabetes developing within 4 wks of adverse nutritional circumstances. Generalized obesity to initiate diabetes is not a prerequisite, although the most metabolically active fat pool (abdominal fat) appears linked to disease onset and terminal energy dynamics during ketosis. The apparent dysfunction in hepatic energy metabolism adds to the comparative appeal of this model. As such, it represents an excellent opportunity for study of nutritional aspects of type 2 diabetes, ie. from diet compostion impacting insulin resistance, to liver and kidney failure and death.

## Competing interests

The authors declare that they have no competing interests.

## Authors' contributions

KCH, FC, AP and EM contributed to various aspects of the design and participated in data collection. FC and AP performed statistical analysis. KCH, AP and FC critically reviewed the paper. FC and KCH interpreted the data and drafted the manuscript. All authors read and approved the final manuscript.

## Supplementary Material

Additional file 1 **Table S1**. Food and water intake, body weight and blood glucose of 8-wk old maleNile rats with different onset to diabetes fed Western-type diet for 24 wks (Expt 3) Click here for file
