# Supplementary material for: Nutritional correlates and dynamics of diabetes in the Nile rat (Arvicanthis niloticus): a novel model for diet-induced type 2 diabetes and the metabolic syndrome
Source: Nutr Metab (Lond). 2010 Apr 15;7:29. doi: 10.1186/1743-7075-7-29 (PMC2868017; doi:10.1186/1743-7075-7-29)
Supplement: Additional file 1 — Table S1. Food and water intake, body weight and blood glucose of 8-wk old maleNile rats with different onset to diabetes fed Western-type diet for 24 wks (Expt 3) [file 1743-7075-7-29-S1.DOC]

**Table S1. Food and water intake, body weight and blood glucose of 8-wk old male**

**Nile rats with different onset to diabetes fed Western-type diet for 24wks (Expt 3)**

|  | |  |  | | | |  | **Western-type diet (SFA:MUFA:PUFA=40:43:17)** | | | | |  |  | |  | |  | | |
| --- | --- | --- | --- | --- | --- | --- | --- | --- | --- | --- | --- | --- | --- | --- | --- | --- | --- | --- | --- | --- |
|  | | **8-12wk of age¥** | | | | | **12-16wk of age** | | | **16-20wk of age** | | | **20-24wk of age** | | | **28-32wk of age** | | | | |
|  | | Early-onset | | | Late-onset | Early-onset | | | Late-onset | Early-onset | Late-onset | | Early-onset | | Late-onset | Early-onset | | | Late-onset | |
|  | |  | | |  | |  | |  |  |  | |  | |  |  | | |  | |
| Body weight (g) | | 103±11 | | | 83±8* | | 115±11 | | 92±6* | 124±12 | 101±5* | | 128±12 | | 114±9 | 129±9 | | | 123±12 | |
|  | |  | | |  | |  | |  |  |  | |  | |  |  | | |  | |
| Body weight gain (g/d) | 1.02±0.15 | | | 0.54±0.16* | | | 0.39±0.10 | | 0.30±0.13 | 0.31±0.15 | 0.31±0.16 | | 0.14±0.18 | | 0.43±0.28* | | 0.07±0.25 | | | 0.08±0.14 |
|  | |  | | |  | |  | |  |  |  | |  | |  |  | | |  | |
| Food intake (kcal/d) | | 34±2 | | | 33±2 | | 31±1 | | 32±2 | 35±3 | 33±2 | | 36±4 | | 31±2* | 45±5 | | | 34±3* | |
|  | |  | | |  | |  | |  |  |  | |  | |  |  | | |  | |
| Growth efficiency (kcal/g gain/d) | | 32±5 | | | 65±18* | | 84±26 | | 126±60 | 145±90 | 170±163 | | 269±91 | | 144±172 | NA† | | | NA† | |
|  | |  | | |  | |  | |  |  |  | |  | |  |  | | |  | |
| Water intake (mL/d) | | 4±1 | | | 6±2 | | 4±1 | | 5±1 | 8±7 | 5±1 | | 14±7 | | 4±1* | 33±16 | | | 16±10* | |
|  | |  | | |  | |  | |  |  |  | |  | |  |  | | |  | |
| Random blood glucose (mg/dL) | | 52±8 | | | 48±10 | | 61±29 | | 48±10 | 150±92 | 47±7* | | 263±48 | | 61±24* | 333±133 | | | 240±152 | |
|  | |  | | |  | |  | |  |  |  | |  | |  |  | | |  | |
|  | |  | | |  | |  | |  |  |  | |  | |  |  | | |  | |
| Values are mean±SD (early-onset n=6, late-onset n=5) | | | | | | | | | |  |  | |  | |  |  | | |  | |
| ^¥^At the start of experiment (at 8wk of age) early-onset rats weighed 72±13g with random blood glucose of 67±27mg/dL,whereas late-onset rats weighed 67±4g with blood glucose of 64±12mg/dL | | | | | | | | | | | | | | | | | | | | |
| † Not analyzed - some rats were losing weight from diabetes | | | | | | | | | | | |  |  | |  |  | | |  | |
| *Significant difference (P<0.05) between early- and late-onset | | | | | | | | | | | |  |  | |  |  | | |  | |
